# Persistent immune imprinting occurs after vaccination with the COVID-19 XBB.1.5 mRNA booster in humans

**DOI:** 10.1016/j.immuni.2024.02.016

**Published:** 2024-03-14

**Authors:** M. Alejandra Tortorici, Amin Addetia, Albert J. Seo, Jack Brown, Kaiti Sprouse, Jenni Logue, Erica Clark, Nicholas Franko, Helen Chu, David Veesler

**Affiliations:** 1 Department of Biochemistry, University of Washington, Seattle, WA 98195, USA; 2 Division of Allergy and Infectious Diseases, University of Washington, Seattle, WA 98195, USA; 3 Howard Hughes Medical Institute, University of Washington, Seattle, WA 98195, USA

**Keywords:** Immune imprinting, SARS-CoV-2, COVID-19 mRNA vaccines, memory B cells, neutralizing antibodies, depletion assays

## Abstract

Immune imprinting describes how the first exposure to a virus shapes immunological outcomes of subsequent exposures to antigenically related strains. SARS-CoV-2 Omicron breakthrough infections and bivalent COVID-19 vaccination primarily recall cross-reactive memory B cells induced by prior Wuhan-Hu-1 spike mRNA vaccination rather than priming Omicron-specific naïve B cells. These findings indicate that immune imprinting occurs after repeated Wuhan-Hu-1 spike exposures, but whether it can be overcome remains unclear. To understand the persistence of immune imprinting, we investigated memory and plasma antibody responses after administration of the updated XBB.1.5 COVID-19 mRNA vaccine booster. We showed that the XBB.1.5 booster elicited neutralizing antibody responses against current variants that were dominated by recall of pre-existing memory B cells previously induced by the Wuhan-Hu-1 spike. Therefore, immune imprinting persists after multiple exposures to Omicron spikes through vaccination and infection, including post XBB.1.5 booster vaccination, which will need to be considered to guide future vaccination.

## eTOC BLURB

Tortorici et al show that the updated XBB.1.5 mRNA COVID booster elicits neutralizing antibodies and memory B cells against current variants that are dominated by recall of pre-existing memory B cells previously induced by Wuhan-Hu-1 spike exposure. Immune imprinting thus persists, including post XBB.1.5 booster vaccination

## Graphical Abstract

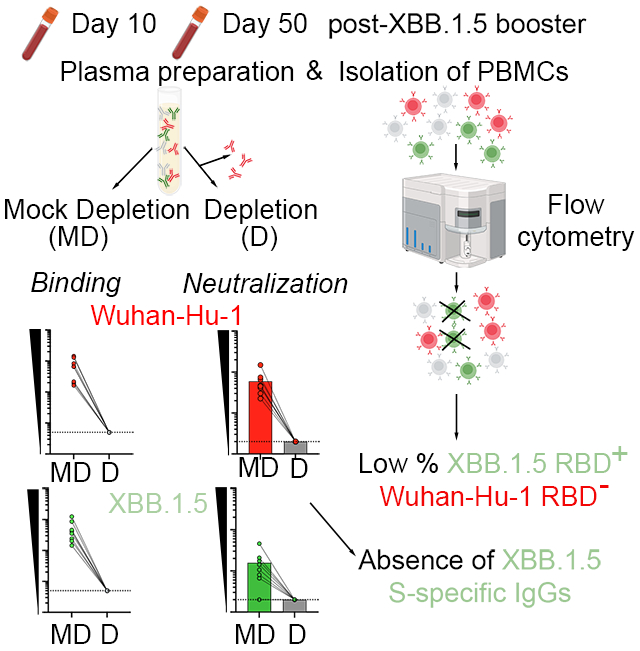

## INTRODUCTION

The SARS-CoV-2 spike (S) glycoprotein promotes entry into host cells and initiate infections^1,2^. S is divided into S_1_ and S_2_ subunits that remain noncovalently linked after proteolytic cleavage during synthesis. In the S_1_ subunit, the receptor-binding domain (RBD) and the N-terminal domain (NTD) recognize the angiotensin-converting enzyme 2 host receptor^1–4^ and attachment factors^5–7^, respectively. The S_2_ subunit is a spring-loaded, metastable fusion machinery which undergoes large-scale conformational changes to fuse the virus and host membranes^8,9^. SARS-CoV-2 S is a main target of antibodies elicited by infection and monoclonal antibodies recognizing specific antigenic sites on the RBD^10–22^, the NTD^23–27^, domain C/SD1^28^ or the fusion machinery^29–35^ inhibit SARS-CoV-2. Furthermore, SARS-CoV-2 serum neutralizing antibody titers are a correlate of protection against COVID-19^36–39^ with most plasma neutralizing activity directed to the S receptor-binding domain (RBD)^16,40,41^.

Numerous safe and effective COVID-19 vaccines have been developed worldwide based on the S glycoprotein or inactivated virus^42–48^. However, continued emergence of SARS-CoV-2 variants harboring tens of amino acid mutations in their S glycoprotein has led to unprecedented evasion from neutralizing antibodies elicited by prior infection or vaccination^12,49–57^. These variants escape clinically authorized monoclonal antibodies and erode the effectiveness of COVID-19 vaccines. Therefore, numerous infection waves occurred globally and were driven by successive evolutionary SARS-CoV-2 lineages, including in individuals who had received multiple COVID-19 vaccine doses^58–60^. As a result, two updated vaccine boosters were developed: a bivalent Wuhan-Hu-1/BA.5 (or Wuhan-Hu-1/BA.1 for a few countries) S mRNA booster vaccine in August 2022^61,62^ and a monovalent XBB.1.5 S mRNA booster vaccine in September 2023^63^.

Recent work showed that antibody responses to Omicron variants are dominated by pre-existing immunity resulting from prior exposure to Wuhan-Hu-1 S, due to immune imprinting^12,55,59,60,64,65^. A study of individuals receiving inactivated Wuhan-Hu-1 viral vaccines, however, found that repeated Omicron infections could overcome immune imprinting, leading to *de novo* elicitation of antibody responses specific for these variants^66^. It is unknown if a similar outcome can be achieved through repeated administration of updated vaccine boosters in individuals previously imprinted via multiple Wuhan-Hu-1 S exposures.

To understand the persistence of immune imprinting, we studied here memory and plasma antibody responses after XBB.1.5 S COVID-19 mRNA booster vaccination. We showed that the XBB.1.5 booster elicited neutralizing antibody responses against past and currently circulating variants that were dominated by recall of pre-existing memory B cells previously induced by Wuhan-Hu-1 S. Our results indicate that immune imprinting persisted after multiple exposures to Omicron S trimers through vaccination and infection, including after administration of the updated XBB.1.5 S booster vaccine, which will influence future vaccination campaigns.

## RESULTS

### XBB.1.5 COVID-19 mRNA booster vaccination elicits broadly neutralizing antibodies against SARS-CoV-2 variants

**Figure 1.**
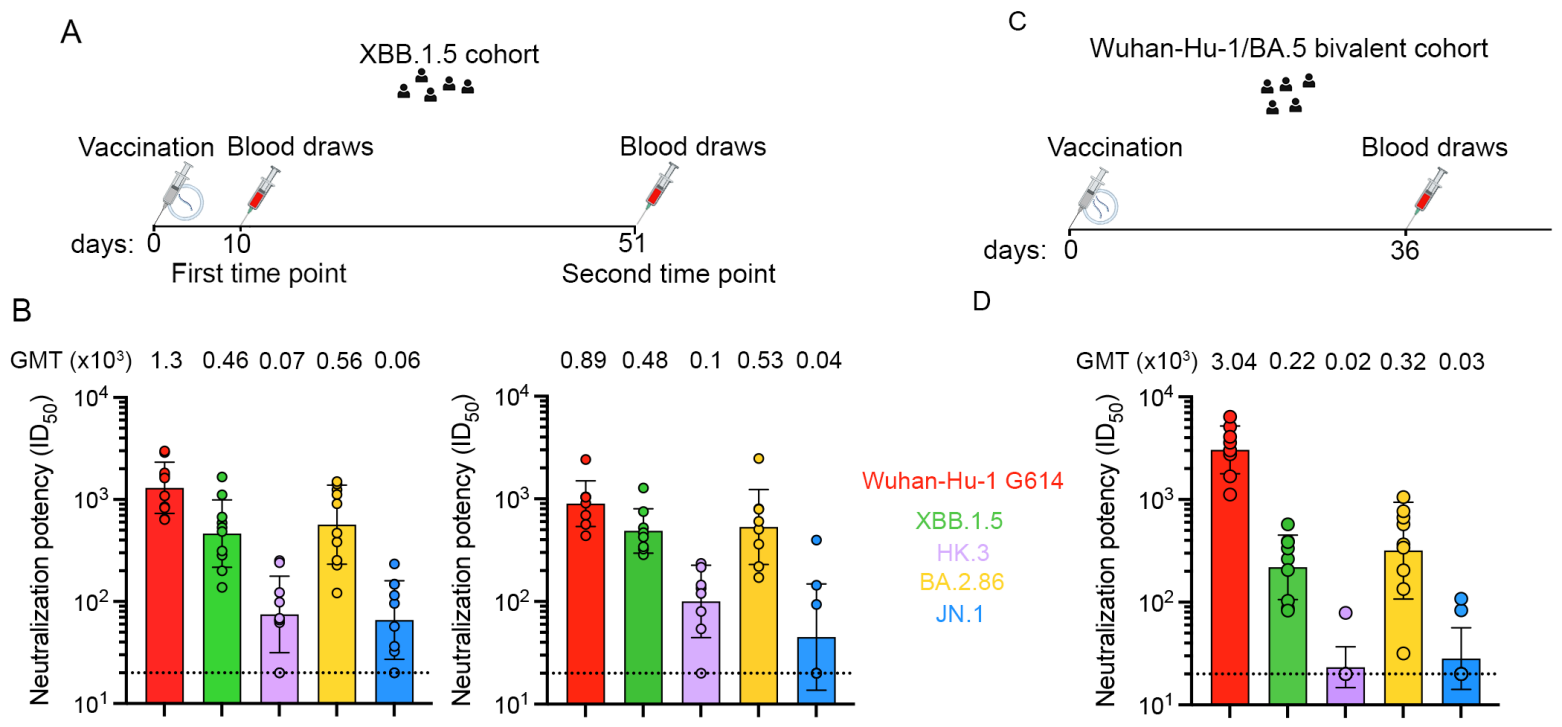
Plasma neutralizing antibody responses elicited by XBB.1.5 S and Wuhan-Hu-1/BA.5 bivalent S mRNA booster vaccination in humans. **A,C,** Timeline of vaccination and blood draws for the XBB.1.5 S (A) and the Wuhan-Hu-1/BA.5 bivalent S (C) mRNA booster vaccine cohorts. **B,D,** Plasma neutralizing antibody titers evaluated using a vesicular stomatitis virus (VSV) pseudotyped with the Wuhan-Hu-1 S harboring the D614G mutation (red), the XBB.1.5 mutations (green), the HK.3 mutation (purple), the BA.2.86 mutations (yellow) or the JN.1 mutation (blue) using plasma obtained 7 to 13 days (mean: 10 days, C left) or 30-63 days (mean: 51 days, C right) after vaccination with the XBB.1.5 S mRNA booster or 22 to 51 days (mean: 36 days) after vaccination with the Wuhan-Hu-1/BA.5 bivalent booster (D). Each data point represents the half-maximal inhibitory dilution (ID_50_) for an individual obtained from averaging two biological replicates with each one comprising two to four technical replicates (excepted for individual 491C for which the ID_50_ values were obtained from four technical replicates for HK.3 and JN.1). For BA.2.86 S VSV neutralization using the plasma samples from the Wuhan-Hu-1/BA.5 bivalent S mRNA booster vaccinated cohort, the ID_50_ values were obtained from one batch of pseudovirus using technical duplicates. Geometric mean titers (GMTs) for each cohort against each pseudotype are indicated above the corresponding bar graph. The dotted dashed lines indicate the limit of detection which is 1/20 for all assays except for neutralizations of Wuhan-Hu-1 G614 S, XBB.1.5 S and BA 2.86 S VSV with XBB.1.5 S booster-elicited vaccinee plasma at day 10 and Wuhan-Hu-1/BA.5 bivalent S booster-elicited vaccinee plasma for which it is 1/40. See also Figures S1 and S2.

To evaluate humoral immunity elicited upon receipt of an XBB.1.5 S mRNA vaccine booster, we collected plasma from individuals who had previously received multiple vaccine doses with or without known infection (Tables 1–2). Plasma samples were obtained at two time points: 7-13 days (mean: 10 days) and 30-63 days (mean: 51 days) following XBB.1.5 S mRNA vaccination (Figure 1A–B, Figure S1). We used a vesicular stomatitis virus (VSV) pseudotyped with the Wuhan-Hu-1/D614G, XBB.1.5, HK.3, BA.2.86 or JN.1 S glycoprotein to assess the potency and breadth of plasma neutralizing antibodies in these cohorts and compare them with plasma collected 21-52 days (mean: 36) following Wuhan-Hu-1/BA.5 S bivalent mRNA S vaccination (Figure 1C–D, Figure S2). Neutralizing activity was highest against the Wuhan-Hu-1/G614 S VSV with geometric mean titers (GMTs) of 1,300 (day 10) and 890 (day 51) after receiving the XBB.1.5 S booster and 3,040 after receiving the Wuhan-Hu-1/BA.5 S bivalent booster. GMTs against XBB.1.5 S VSV were 460 (day 10) and 480 (day 51) after receiving the XBB.1.5 S booster and 220 for bivalent vaccinees. Neutralization of the XBB.1.5 descendant HK.3 variant, which harbors the RBD L455F/F456L flip mutations^67^, was also higher after administration of the XBB.1.5 S booster (GMTs 70 and 100 at days 10 and 51, respectively) relative to the bivalent booster (GMT 20). XBB.1.5 S vaccination induced neutralizing antibody titers of 560 (day 10) and 530 (day 51) against BA.2.86 S VSV and of 60 (day 10) and 40 (day 51) against JN.1, respectively. Wuhan-Hu-1/BA.5 S bivalent vaccination elicited respective GMTs of 320 and 30 against BA.2.86 and JN.1. These data show that the updated XBB.1.5 S mRNA vaccine booster elicits greater neutralizing antibody responses against a panel of recently and currently circulating variants, including mismatched pseudoviruses, relative to the previous Wuhan-Hu-1/BA.5 S bivalent booster, which is expected to translate into enhanced protection in the real world.

### XBB.1.5 COVID-19 mRNA booster vaccination recalls Wuhan-Hu-1 S cross-reactive antibodies

**Figure 2.**
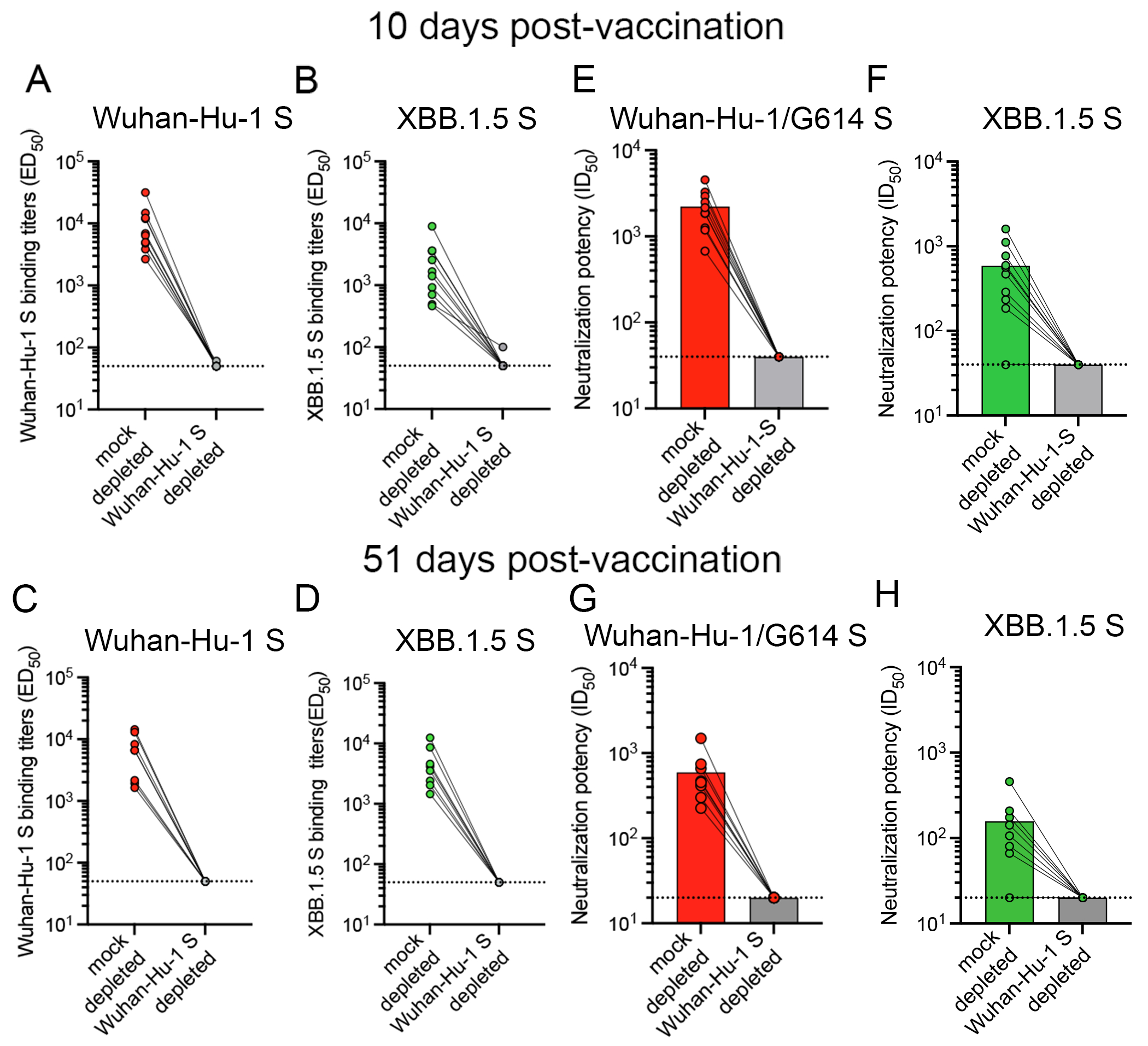
Depletion of Wuhan-Hu-1 S-reactive plasma antibodies abrogates XBB.1.5 S binding and neutralizing antibody titers. **A-D,** Antibody binding titers (expressed as half-maximal effective dilution, ED_50_) against Wuhan-Hu-1 S (**A,C**) and XBB.1.5 S (**B,D**) in XBB.1.5 S booster-elicited vaccinee plasma that were either mock-depleted (left) or depleted (right) of antibodies recognizing Wuhan-Hu-1 S as determined by ELISA. **E-H,** Neutralizing antibody titers against Wuhan-Hu-1 S VSV (**E,G**) and XBB.1.5 S VSV (**F, H**) in XBB.1.5 S booster-elicited vaccinee plasma that were mock-depleted (left) or depleted (right) from antibodies recognizing Wuhan-Hu-1 S. Samples were collected 7-13 days (mean: 10 days, A-B and E-F) or 30-63 days (mean: 51 days, C-D and G-H) after receiving the XBB.1.5 S mRNA booster. The dotted lines indicate the limit of detection of 1/50 for the ELISA and 1/40 or 1/10 for the neutralization assays. In panels A-D, each data point represents the half-maximal effective dilution (ED_50_) for an individual obtained from averaging technical duplicates from one representative out of 2 biological replicates. In panels E-H, each data point represents the half-maximal inhibitory dilution (ID_50_) for an individual obtained from the average of two biological experiments each done with technical duplicates which were averaged. Geometric mean titers (GMTs) for each cohort against each pseudovirus are shown as bar graphs. See also Figures S3 and S4.

The finding that administration of an XBB.1.5 S booster elicited higher plasma neutralizing activity against Wuhan-Hu-1/D614G S VSV (vaccine-mismatched) relative to XBB.1.5 S VSV (vaccine-matched) at both time points examined is a serological indication of immune imprinting. To investigate this further, we depleted polyclonal plasma antibodies recognizing the Wuhan-Hu-1 S trimer and assessed binding titers against the Wuhan-Hu-1 S and XBB.1.5 S ectodomain trimers using ELISAs. As expected, no antibodies binding to Wuhan-Hu-1 S were detected after depletion. Moreover, depletion of antibodies targeting Wuhan-Hu-1 S abrogated binding to XBB.1.5 S at 10 days and 51 days post XBB.1.5 S booster vaccination (Figure 2A–D, Figure S3). Accordingly, we did not detect neutralizing antibodies against Wuhan-Hu-1/D614G S VSV and XBB.1.5 S VSV following depletion (Figure 2E–H, Figure S4), indicating the absence of XBB.1.5 S-specific antibodies in the plasma of these subjects at all time points (i.e. that were not cross-reactive with Wuhan-Hu-1 S). These data suggest that XBB.1.5 S vaccination boosts cross-reactive plasma antibody titers previously elicited by Wuhan-Hu-1 S exposure, which are also binding to and neutralizing XBB.1.5 and other variants instead of inducing *de novo* antibody responses against XBB.1.5 S.

### XBB.1.5 COVID-19 mRNA booster vaccination primarily recalls Wuhan-Hu-1 RBD-directed memory B cells

**Figure 3.**
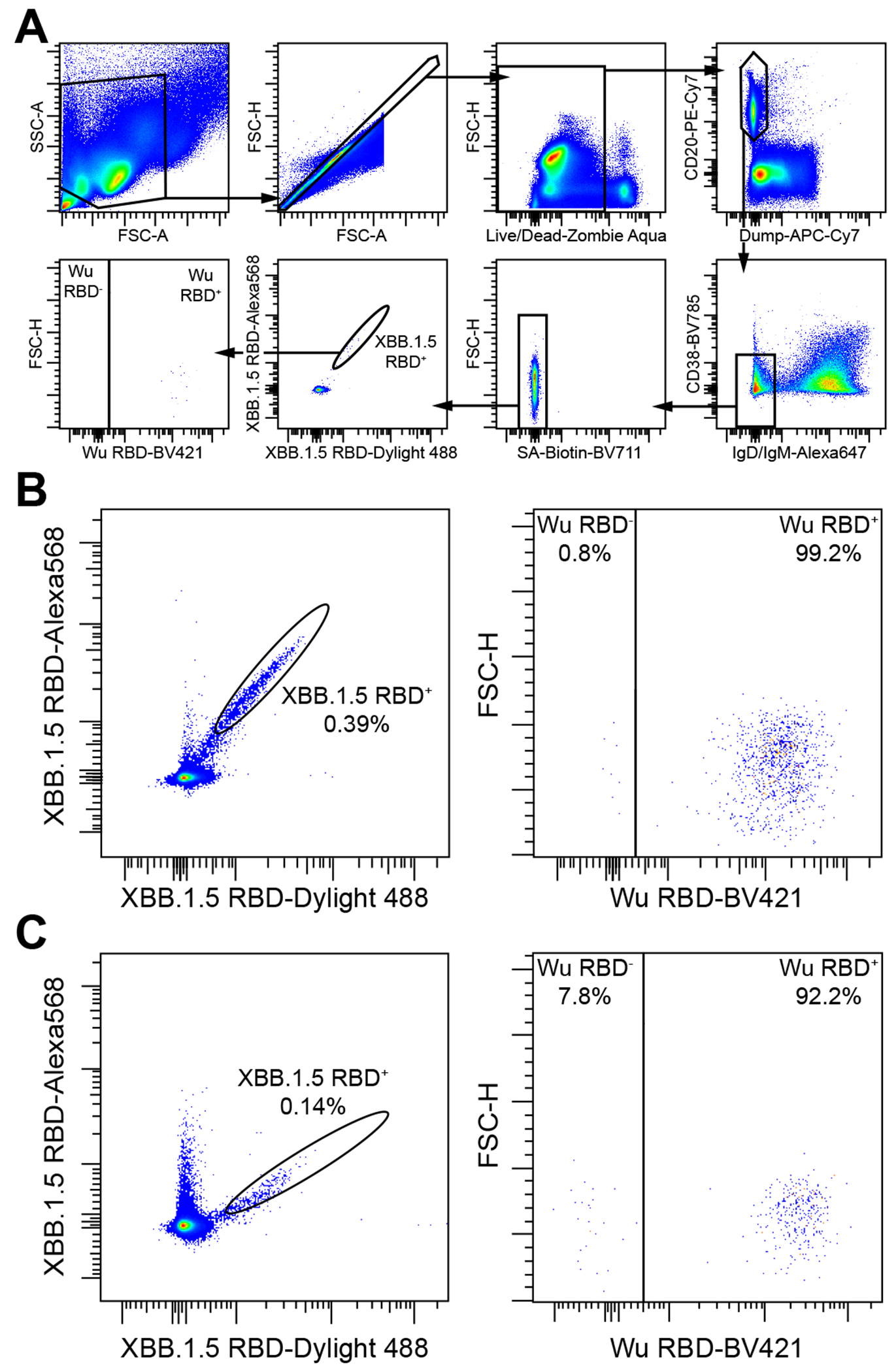
Wuhan-Hu-1 S-reactive memory B cells dominate humoral immune responses elicited by XBB.1.5 S mRNA booster vaccination in humans. **A,** Gating strategy to evaluate the cross-reactivity with the Wuhan-Hu-1 RBD of XBB.1.5 RBD+ memory B cells. Dump includes markers for CD3, CD8, CD14, and CD16. **B-C,** XBB.1.5 RBD double positive memory B cells (left) were analyzed for cross-reactivity with the Wuhan-Hu-1 (Wu) RBD (right). Memory B cells were obtained from peripheral blood collected from individuals 7-13 days (mean: 10 days, B) or 30-63 days (mean: 51 days, C) after receiving the XBB.1.5 S mRNA booster using flow cytometry. See also Figure S5.

We subsequently analyzed memory B cell populations found in the peripheral blood at the same time points following XBB.1.5 S vaccination by measuring the frequency of XBB.1.5 RBD-reactive memory B cells which also bound to the Wuhan-Hu-1 RBD using flow cytometry cytometry (Figure 3A) Only 5 out of the 12 individuals profiled had memory B cells that recognized the XBB.1.5 RBD, but not the Wuhan-Hu-1 RBD, and these memory B cells were rare (0.4-13.3% individual frequencies) at 10 days post XBB.1.5 S booster vaccination (Figure 3B, Figure S5 and Table 1). Consistent with the first time point, memory B cells recognizing the XBB.1.5 RBD, without cross-reacting with the Wuhan-Hu-1 RBD, remained rare (4.9-18.8% individual frequencies) and were identified for only 3 out of 9 individuals at 51 days post XBB.1.5 S booster vaccination (Figure 3C, Figure S5 and Table 1). We observed a modest increase in frequency of XBB.1.5 RBD-specific memory B cells for only 2 of the 7 individuals who contributed samples at both timepoints: 0% to 4.9% for 106H and 13.3% to 18.8% for 385C. *De novo* elicitation of memory B cells is thus possible, as observed here for the variant-specific XBB.1.5 S mRNA booster, but difficult to induce due to preferential recall of pre-existing memory B cells given that the vast majority of XBB.1.5 RBD-binding memory B cells also recognize the Wuhan-Hu-1 RBD.

## DISCUSSION

The lack of detectable plasma antibodies specific for XBB.1.5 S and the scarcity of memory B cells binding to the XBB.1.5 RBD, but not the Wuhan-Hu-1 RBD, indicate that the humoral immune responses elicited by XBB.1.5 S vaccination are dominated by recall of pre-existing memory B cells previously induced by Wuhan-Hu-1 S vaccination instead of inducing *de novo* responses against this new variant. These findings held true for at least ~2 months post vaccination and concur with observations made after Omicron BA.1, BA.2 and BA.5 breakthrough infections^12,60,68^ and with that made after the roll out of the bivalent Wuhan-Hu-1/BA.5 and Wuhan-Hu-1/BA.1 S vaccine boosters^55,64^.

Prior work showed that two booster vaccinations with Omicron S or two Omicron infections were sufficient to induce broad serum neutralizing activity and elicit strong memory B cell responses specific for Omicron RBDs in mice and humans primed with inactivated SARS-CoV-2 Wuhan-Hu-1 vaccines^66^. Although comparative studies are needed to understand the apparent reduced imprinting of inactivated vaccines, as compared to mRNA vaccines, the stronger humoral immune responses elicited by the latter vaccines may partly explain these differences although the total number of exposures are markedly distinct across cohorts studied^40,55,60,65,66,69–71^. As a result, individuals who received an mRNA vaccine primary series may possess a higher frequency of memory B cells recognizing pan-variant epitopes, many of which may be non- or weakly neutralizing, relative to subjects receiving an inactivated viral vaccine primary series. These cross-reactive memory B cells may be reactivated rapidly upon exposure to SARS-CoV-2 variant S proteins^72^, reducing the ability to mount de novo B cell responses in mRNA vaccinees. In contrast, the possibly lower frequency of cross-reactive memory B cells in subjects primed with inactivated vaccines, relative to mRNA vaccinees, may allow for more efficient de novo recruitment of B cells recognizing variant-specific epitopes, as induced by updated vaccination or infection with SARS-CoV-2 variants.

Collectively, our results indicate that the updated XBB.1.5 S vaccine elicit neutralizing antibodies against circulating variants and point to the persistence of immune imprinting which will need to be considered to guide the design of future vaccine boosters in populations with repeated Wuhan-Hu-1 S exposures.

### Limitations of the study

We analyzed samples from cohorts recruited in the Seattle area, which include subjects with a high number of Wuhan-Hu-1 S exposures. Our cohorts are inherently limited in size although recent findings made from different cohorts validate our conclusions^73^. Furthermore, as SARS-CoV-2 S-specific memory B cells continue to evolve and increase in frequency for several months post infection or vaccination^74–78^, future analysis at much later time points (6-12 months) will shed light on long-term immunological impact of imprinting.

### Resource availability

#### Lead contact

Further information and requests for resources and reagents should be directed to and will be fulfilled by the lead contact David Veesler (dveesler@uw.edu)

### Materials availability

Requests for reagents and resources generated in this study will be fulfilled by the lead contact.

### Data and code availability

All the data are presented in this manuscript.

This paper does not report original code.

Any additional information required to reanalyze the data reported is available from the lead contact upon request.

### Experimental model and study participant details

#### Cell lines

VeroE6-TMPRSS2 (geneticin resistant) is a female cell line (obtained from JCRB-Cell Bank), VeroE6-TMPRSS2 puromycin resistant cells, generated using a lentivirus system^5^, is a female cell line, Expi293F is a female cell line (obtained from ThermoFisher Scientific). HEK293T is a female cell line (obtained from ATCC) and Il-mouse hybridoma is a female cell line (obtained from ATCC). Cells were cultivated at 37°C in an atmosphere of 5 % CO_2_ for adherent cells and 8% CO_2_ with 130 rpm of agitation for suspension cells. None of the cell lines used were routinely tested for mycoplasma contamination.

#### Sample donors and collection

Plasma and PBMCs were collected after informed consent from participants in the prospective longitudinal Hospitalized or Ambulatory Adults with Respiratory Viral Infections (HAARVI) study from Washington State, USA, which was approved by University of Washington Institutional Review Board (protocol #STUDY00000959). Demographics information is provided in Tables 1 and 2.

#### Plasmids

Plasmids encoding Wuhan-Hu-1 S Hexapro ectodomain (residues 1-1208) and XBB.1.5 S Hexapro ectodomain (residues 1-1203) were synthesized into pCDNA 3.1 (−) and pcDNA 3.1 (+), respectively. Both genes were synthesized by Genscript and harbor the HexaPro mutations^79^, a wildtype signal peptide, a furin cleavage site mutated _685_RSV_687_ to _685_SSV_687_, an avi-tag, and an octa-his tag for affinity purification.

Constructs for membrane-anchored S glycoproteins from SARS-CoV-2 Wuhan-Hu-1/G614, XBB.1.5 (mutations relative to Wuhan-Hu-1/G614: T19I, L24del, P25del, P26del, A27S, V83A, G142D, Y144del, H146Q, Q183E, V213E, G252V, G339H, R346T, L368I, S371F, S373P, S375F, T376A, D405N, R408S, K417N, N440K, V445P, G446S, N460K, S477N, T478K, E484A, F486P, F490S, Q498R, N501Y, Y505H, H655Y, N679K, P681H, N764K, D796Y, Q954H, N969K), HK.3 (XBB.1.5 mutations with L455F and F456L), BA.2.86 (mutations: 17M, L18P, T19L, F20ins, T20N, R21L, T22I, Q23T, L24T, P25T, P26Q ,A27S, S50L, H69del, V70del, V127F, G142D, Y144del, F157S, R158G, N211del, L212I, V213G, L216F, H245N, A264D, I332V, G339H, K356T, S371F, S373P, S375F, T376A, R403K, D405N, R408S, K417N, N440K, V445H, G446S, N450D, L452W, N460K, S477N, T478K, N481K, V483del, E484K, F486P, Q498R, N501Y, Y505H, E554K, A570V, P621S, H655Y, I670V, N679K, P681R, N764K, D796Y, S939F, Q954H, N969K, P1143L) and JN.1 (BA.2.86 mutations with L455S) contain the wild-type signal peptide and a 21-amino acid C-terminal deletion^80^ with no tags. All genes were synthesized by Genscript and cloned into an HDM vector, inserted in frame with a Kozak sequence to direct translation and codon optimized for expression in mammalian cells. Plasmids encoding the SARS-CoV-2 Wuhan-Hu-1 and the XBB.1.5 RBDs were previously described and their boundaries are N-_328_RFPN_331_ and C-_527_KKST_531_^1,55^. Moreover, the SARS-CoV-2 Wuhan-Hu-1 RBD construct contains an N-terminal mu-phosphatase signal peptide and C-terminal octa-histidine tag followed by an avi-tag. The XBB.1.5 RBD construct contains an N-terminal BM40 signal peptide and a C-terminal octa-histidine tag followed by an avi-tag.

#### Recombinant protein production

SARS-CoV-2 Wuhan-Hu-1 and XBB.1.5 HexaPro S ectodomains were expressed in Expi293 cells at 37°C and 8% CO_2_. Cells were transfected with the corresponding plasmids using Expifectamine following the manufacturer’s instructions. Four days post-transfection, supernatants were clarified by centrifugation at 4,121g for 30 minutes, supplemented with 25 mM phosphate pH 8.0, 300 mM NaCl, and 0.5 mM phenylmethylsulfonyl fluoride (PMSF). Supernatant was then 0.22μm vacuum filtered and passed through 1 mL His trap HP or His-Trap Excel column (Millipore, Sigma) previously equilibrated in 25 mM phosphate pH 8.0, 300 mM NaCl. S proteins were eluted using a buffer identical to the binding buffer with the addition of 300 mM imidazole. Fractions containing the proteins were pooled and buffer exchanged to 50 mM Tris-HCl pH 8.0, 150 mM NaCl using a centrifugal filter device with a MWCO of 100 kDa and stored at 4°C or immediately used.

The SARS-CoV-2 Wu and XBB.1.5 RBDs were expressed and purified as described above. Following buffer exchange using a centrifugal filter with a MWCO of 10kDa, the purified RBDs were biotinylated using the BirA biotin-protein ligase reaction kit (Avidity). The biotinylated proteins were passed, washed, and eluted again on the same affinity column, concentrated and ran over a Superdex200 increase 10/300 size-exclusion column (Cytiva) equilibrated in 50mM Tris pH 8.0 and 150mM NaCl or 20mM Phosphate pH 8.0 and 100mM NaCl. Fractions corresponding to monomeric and monodisperse RBDs were collected, flash frozen, and stored at −80°C until use.

#### Plasma antibody depletion

Invitrogen His-Tag Dynabeads (ThermoFisher 10104D) were used for depletion of plasma samples from antibodies recognizing the Wuhan-Hu-1 S trimer, as previously described^40^ with some modifications. Vortexed beads were incubated at room temperature on an Invitrogen DynaMag-2 Magnet (ThermoFisher 12–321-D) for two minutes to allow beads to separate for the liquid phase. Supernatant was discarded and beads were washed one time with TBS-T (20 mM Tris-HCl pH7.5, 150 mM NaCl, 0.1% (w/v) Tween 20) and divided in two tubes. After a two-minute incubation on the magnet, TBS-T supernatants were discarded and one set of beads was incubated with 4 mg of purified his-tagged SARS-CoV-2 Wuhan-Hu-1 S ectodomain trimer in purification buffer (Wuhan-Hu-1 S depletion) and the other set was incubated with TBS-T alone (mock depletion) with gentle rotation for 1 h at room temperature. Supernatants were discarded using the magnet and beads were washed three times with TBS-T. Subsequently, 20 μl of each of the plasma samples were incubated with 80 μl of S-loaded beads or mock-loaded beads for 1 h at 37°C during which time they were mixed every 15 min. Plasma samples were separated from the beads using the magnet and subjected to a second round of depletion using the same protocol as described for the first round. incubated Supernatants were recovered using a magnet to separate them from the beads and used for neutralization and binding assays.

#### VSV pseudotyped virus production

Vesicular stomatitis virus (VSV) were pseudotyped with SARS-CoV-2 Wuhan-Hu-1 S harboring the G614, XBB.1.5, HK3.1, BA.2.86 or JN.1 mutations following a previously described protocol^81^. Briefly, HEK293T cells seeded in poly-D-lysine-coated 10-cm dishes in DMEM supplemented with 10% FBS and 1% PenStrep were transfected with 24 μg of the corresponding plasmid, 60 μl Lipofectamine 2000 (Life Technologies) in 5 ml of Opti-MEM, following the manufacturer’s instructions. After 5 h at 37°C, 5 ml of DMEM supplemented with 20% FBS and 2% PenStrep were added. The next day, cells were washed three times with DMEM and were transduced with VSVΔG-luc^82^. After 2 h, the virus inoculum was removed and cells were washed five times with DMEM prior to the addition of DMEM supplemented with anti-VSV-G antibody [Il-mouse hybridoma supernatant diluted 1 to 25 (v/v), from CRL-2700, ATCC] to minimize parental background. After 18-24 h, supernatants containing pseudotyped VSV were harvested, centrifuged at 2,000 x g for 5 min to remove cellular debris, filtered with a 0.45 μm membrane, concentrated 10 times using a 30 kDa cut off membrane (Amicon), aliquoted, and frozen at −80°C.

#### Pseudotyped VSV neutralization

VeroE6-TMPRSS2 cells were seeded into coated clear bottom white walled 96-well plates at 40,000 cells/well and cultured overnight at 37°C. Eleven 3-fold serial dilutions of each plasma sample were prepared in DMEM. Pseudotyped VSV viruses, diluted 1 to 20 in DMEM containing anti-VSV-G antibody, were added 1:1 (v/v) to each plasma sample dilution and mixtures of 50 μl volume were incubated for 45-60 min at 37°C. VeroE6-TMPRSS2 cells were washed three times with DMEM and 40 μL of the mixture containing pseudotyped virus and plasma samples were added. Two hours later, 40 μL of DMEM were added to the cells. After 17-20 h, 70 μL of One-Glo-EX substrate (Promega) were added to each well and incubated on a plate shaker in the dark at 37°C. After 5-15 min incubation, plates were read on a Biotek Neo2 plate reader. Measurements were done in duplicate or triplicate with at least two biological replicates. Relative luciferase units were plotted and normalized in Prism (GraphPad): cells without pseudotyped virus added were defined as 0 % infection or 100 % neutralization, and cells with virus only (no plasma) were defined as 100 % infection or 0 % neutralization.

#### Enzyme-linked immunosorbent assays (ELISA)

Analysis of plasma binding antibodies for samples mock-depleted or depleted of antibodies binding to the Wuhan-Hu-1 S ectodomain trimer was performed using ELISAs. Briefly, clear flat bottom Immuno Nonsterile 384-well plates (Thermo Scientific) were coated overnight at room temperature with 30 μl of Wuhan-Hu-1 S or XBB.1.5 S prepared at 3 μg/ml in PBS (137 mM of NaCl, 2.7 mM of KCl, 10 mM of Na2HPO4, and 1.8 mM of KH2PO4, pH 7.2). The next day, plates were blocked with Blocker^™^ Casein (Thermo Scientific) and subsequently incubated with serial dilutions of plasma samples for 1 h at 37°C. After four washing steps with TBS-T, goat anti-human IgG-Fc secondary antibody conjugated to HRP (Invitrogen A18817, diluted 1/500) was added and incubated for 1 h at 37°C. Plates were washed four times with TBS-T and KPL SureBlue Reserve^™^ TMB Microwell Peroxidase Substrate (VWR) was added. After 2 min incubation, 1N HCl was added and absorbance at 405 nm was measured using a Biotek Neo2 plate reader. Data were plotted using Prism (GraphPad).

#### Flow cytometry analysis of SARS-CoV-2 RBD-reactive memory B cells

To define specific B cell populations reactive with the XBB.1.5 and the Wuhan-Hu-1 RBDs, RBD–streptavidin tetramers conjugated to fluorophores were generated by incubating biotinylated RBDs with streptavidin at a 4:1 molar ratio for 30 min at 4 °C. Excess of free biotin was then added to the reaction to bind any unconjugated sites in the streptavidin tetramers. The RBD-streptavidin tetramers were washed once with PBS (137 mM NaCl, 2.7 mM KCl, 10 mM Na_2_HPO_4_, 1.8 mM KH_2_PO_4_, pH 7.4) and concentrated with a 100-kDa cut-off centrifugal concentrator (Amicon). An additional streptavidin tetramer conjugated to biotin only was generated and included in the staining as decoy.

Approximately 5 to 15 million PMBCs were collected 7-13 days and 30-63 days post-vaccination for individuals who received an XBB.1.5 S mRNA vaccine booster. Cells were collected by centrifugation at 400g for 2 mins at 4°C, washed twice with PBS and stained with Zombie Aqua dye (Biolegend) diluted 1:100 in PBS at room temperature. After 30 min incubation, cells were washed twice with PBS and stained with antibodies for CD20-PECy7 (BD), CD3-Alexa eFluor780 (Thermo Fisher), CD8-Alexa eFluor780 (Thermo Fisher), CD14-Alexa eFluor780 (Thermo Fisher), CD16-Alexa eFluor780 (Thermo Fisher), IgM-Alexa Fluor 647 (BioLegend), IgD-Alexa Fluor 647 (BioLegend), and CD38-Brilliant Violet 785 (BioLegend), all diluted 1:200 in Brilliant Stain Buffer (BD), along with the RBD-streptavidin tetramers for 30 min at 4°C. Cells were washed three times, resuspended in PBS, and passed through a 35-μm filter before being examined on a BD FACSymphony A3 for acquisition and FlowJo 10.8.1 for analysis. Gates for identifying the XBB.1.5 RBD double-positive population as well as the subsequent Wuhan-Hu-1 RBD-positive and Wuhan-Hu-1 RBD-negative populations were drawn based on staining of fluorescent minus one controls.

## Supplementary Material

FigS1

FigS2

FigS3

FigS4

FigS5

Supp Figure Legends

## Figures and Tables

**Table 1.** Demographics information of the subjects in the XBB.1.5 vaccinee cohorts.

Patient ID	Sex	Age	Number of pre-Omicron SARS-CoV-2 infection (before 11/2021)	Number of post-Omicron SARS-CoV-2 infection (after 11/2021)	Number of Wuhan-Hu-1 vaccine doses	Number of bivalent Wuhan-Hu-1/BA.5 vaccine doses	Number of XBB.1.5 vaccine doses	Days between XBB.1.5 vaccine and blood draw
48H	Female	40	0	0	3	1	1	7
106H	Male	40	0	0	3	1	1	7, 63
108C	Female	67	1	1	4	2	1	8, 55
117C	Female	71	1	0	4	1	1	10, 45
205C	Male	81	2	0	4	2	1	42
245C	Female	31	1	1	3	1	1	9
334C	Female	27	0	1	3	1	1	13, 56
385C	Female	30	1	0	3	1	1	11, 53
434C	Female	56	0	1	4	1	1	11,62
446C	Female	60	0	1	4	0	1	13
471C	Male	71	0	1	4	1	1	30
473C	Female	61	0	1	4	1	1	49
485C	Female	41	0	1	4	1	1	11
491C	Female	55	0	1	4	1	1	10

**Table 2.** Demographics information of the subjects in the Wuhan-Hu-1/BA.5 bivalent vaccinee cohorts.

Patient ID	Sex	Age	Number of pre-Omicron SARS-CoV-2 infection (before 11/2021)	Number of post-Omicron SARS-CoV-2 infection (after 11/2021)	Number of Wuhan-Hu-1 vaccine doses	Number of bivalent Wuhan-Hu-1/BA.5 vaccine doses	Number of XBB.1.5 vaccine doses	Days between bivalent Wuhan-Hu-1/BA.5 vaccine and blood draw
**318C**	Female	**20**	**0**	**1**	**3**	**1**	**0**	**43**
**319C**	Male	**24**	**0**	**1**	**3**	**1**	**0**	**29**
**392C**	Male	**74**	**0**	**1**	**5**	**1**	**0**	**41**
**400C**	Female	**21**	**0**	**1**	**3**	**1**	**0**	**23**
**403C**	Female	**21**	**0**	**1**	**3**	**1**	**0**	**34**
**414C**	Male	**20**	**0**	**1**	**3**	**1**	**0**	**52**
**422C**	Female	**20**	**0**	**1**	**3**	**1**	**0**	**37**
**424C**	Female	**21**	**0**	**1**	**3**	**1**	**0**	**30**
**445C**	Female	**25**	**0**	**1**	**3**	**1**	**0**	**37**

**Table:** Key Resources Table

REAGENT or RESOURCE	SOURCE	IDENTIFIER
**Bacterial Strains**		
*E. coli* DH10B Competent Cells	Invitrogen	Cat# 44-0099
**Experimental Models: Cell Lines**		
HEK293T cells	ATCC	Cat# CRL-11268
Expi293F cells	ThermoFisher Scientific	Cat# A14527
VERO-TMPRSS2 cells (geneticin resistant)	JCRB-Cell Bank	Cat# JCRB1819
VERO-TMPRSS2 cells (puromycin resistant)	Lempp et al., 2021	DOI: 10.1038/s41586-021-03925-1.
Il-mouse hybridoma	ATCC	CRL-2700
**Recombinant DNA**		
pcDNA3.1(−): Wuhan-Hu-1 S Hexapro ectodomain		This study
pcDNA3.1(+): XBB.1.5 S Hexapro ectodomain	Genescript	This study
HDM vector: Wuhan-Hu-1/G614 Full-length	Bowen et al., 2022	DOI: 10.1126/science.abq0203
HDM vector: XBB.1.5 S Full-length	Genescript	This study
HDM vector: BA.2.86 S Full-length	Genescript	This study
HDM vector: HK.3.1 S Full-length	Genescript	This study
HDM vector: JN.1 S Full-length	Genescript	This study
pcDNA3.1(−): Wuhan-Hu-1 RBD	Walls et al, 2020	DOI:https://doi.org/10.1016/j.cell.2020.02,058
pcDNA3.1(+): XBB.1.5 RBD	Addetia et al, 2023	https://doi.org/10.1038/s41586-023-06487-6
**Kits, reagents and materials**		
BirA biotin-protein ligase standard reaction kit	Avidity	Cat# 341113
poly-D-lysine-coated 10 cm dishes		
Immuno Nonsterile 384-well plates	ThermoFisher	Cat# 464718
Blocker^™^ Casein	ThermoFisher	Cat# J60289.AP
Lipofectamine 2000 Transfection Reagent	Invitrogen	Cat# 11668027
SureBlue Reserve^™^ TMB Microwell Peroxidase Substrate, KPL	VWR	Cat# 5120-0083
Bio-Glo^™^ Luciferase Assay System	Promega	Cat# G7940
ExpiFectamine™ 293 Transfection Kit	ThermoFisher	Cat# A14525
EndoFree Plasmid Mega kit	Qiagen	Cat# 12381
QIAprep Spin Miniprep Kit	Qiagen	Cat# 27106X4
His-Trap High Performance	Millipore Sigma	Cat#Cityva29091596
His-Trap Excel	Millipore Sigma	Cat#Cityva29048586
His-Tag Dynabeads	ThermoFisher	Cat# 10104D
DynaMag-2 Magnet	ThermoFisher	Cat# 12–321-D
VSVΔG-luc DNA	Kaname et al., 2010	DOI: 10.1128/JVI.02519-09
100-kDa cut-off concentrator	Amicon	Cat# UFC910024
Zombie Aqua dye	Biolegend	Cat# 423101
CD20-PECy7	BD Biosciences	Cat# 335793
CD3-Alexa eFluor780	ThermoFisher	Cat# 47-0037-41
CD8-Alexa eFluor780	ThermoFisher	Cat# 47-0086-42
CD14-Alexa eFluor780	ThermoFisher	Cat# 47-0149-42
CD16-Alexa eFluor780	Thermo Fisher	Cat# 47-0168-41
IgM-Alexa Fluor 647	BioLegend	Cat# 314535
IgD-Alexa Fluor 647	BioLegend	Cat# 348227
CD38-Brilliant Violet 785	BioLegend	Cat# 303529
Brilliant Stain Buffer	BD Biosciences	Cat# 563794
**Software and Algorithms**		
Prism 9	GraphPad Software	https://www.graphpad.com/scientific-software/prism/
BioRender	N/A	https://www.biorender.com
FlowJo 10.8.1	BD Biosciences	https://www.flowjo.com/

## HIGHLIGHTS

XBB.1.5 mRNA vaccine elicits neutralizing antibodies against current variants

Depletion of Wuhan-Hu-1 S-reactive plasma antibodies abrogate XBB.1.5 neutralization

XBB.1.5 mRNA vaccine primary recalls Wuhan-Hu-1 S-reactive memory B cells
