# Supplementary material for: Persistent immune imprinting occurs after vaccination with the COVID-19 XBB.1.5 mRNA booster in humans
Source: Immunity. Author manuscript; Available in PMC 2025 Aug 18. (PMC12360627; doi:10.1016/j.immuni.2024.02.016)
Supplement: FigS1 [file NIHMS2101333-supplement-FigS1.pdf]

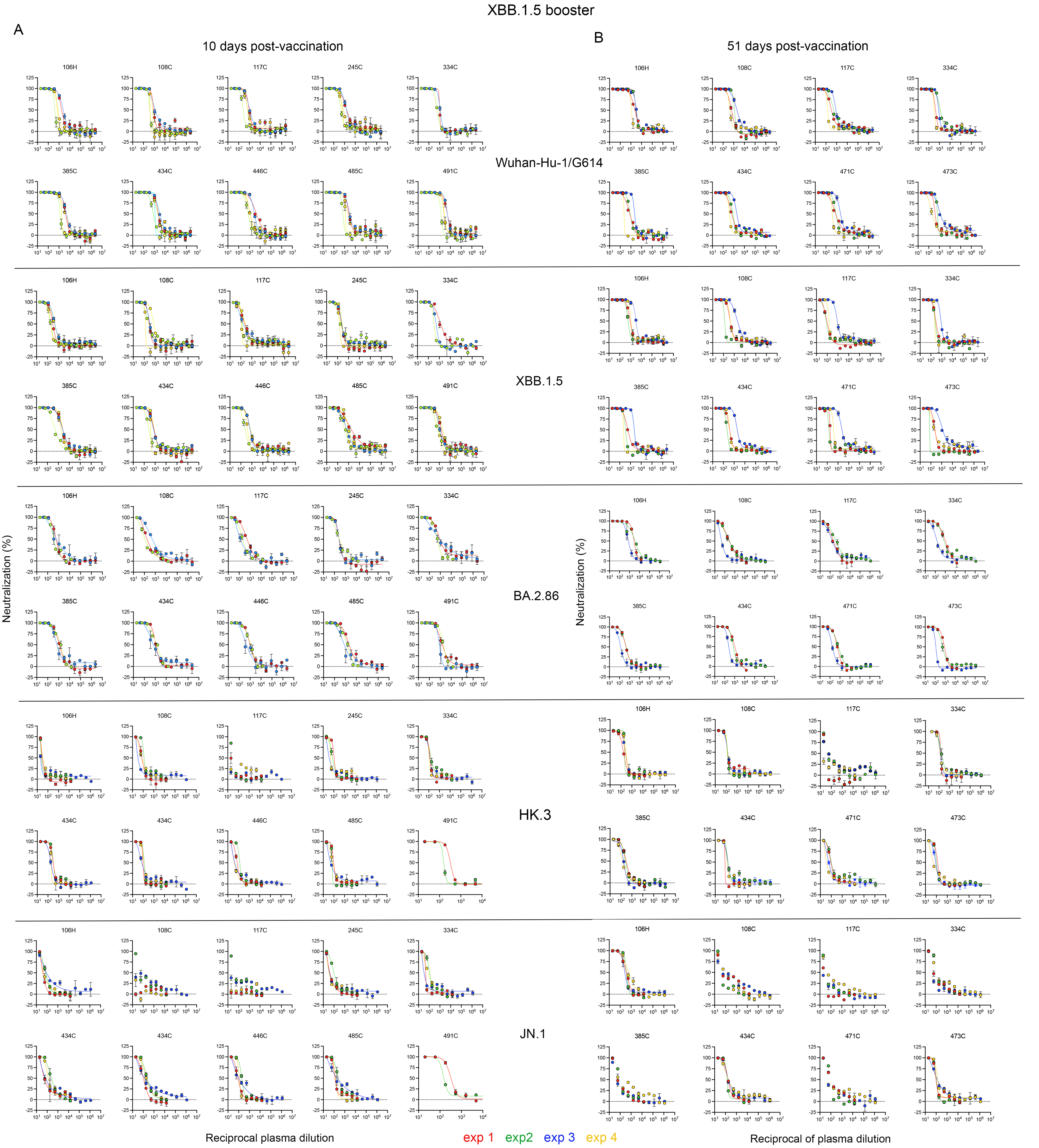

XBB.1.5 booster
A
10 days post-vaccination
B
51 days post-vaccination
Wuhan-Hu-1/G614
XBB.1.5
BA.2.86
HK.3
JN.1
106H
108C
117C
245C
334C
385C
434C
446C
485C
491C
471C
473C
Neutralization (%)
Reciprocal plasma dilution
Reciprocal of plasma dilution
exp 1
exp2
exp 3
exp 4
