# Supplementary material for: Persistent immune imprinting occurs after vaccination with the COVID-19 XBB.1.5 mRNA booster in humans
Source: Immunity. Author manuscript; Available in PMC 2025 Aug 18. (PMC12360627; doi:10.1016/j.immuni.2024.02.016)

Bivalent Wuhan-Hu-1/BA.5 booster

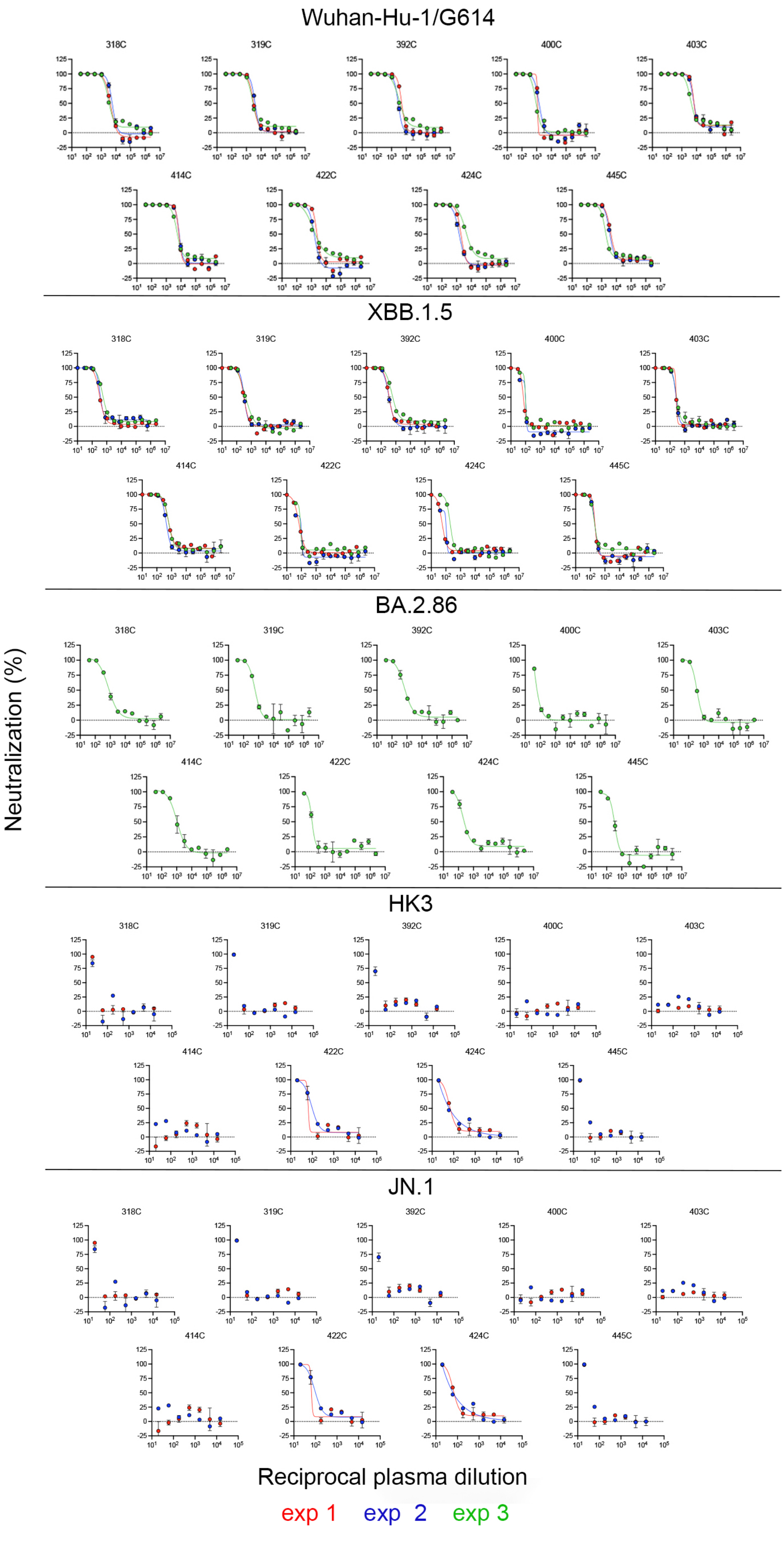

Supplement: FigS2 [file NIHMS2101333-supplement-FigS2.pdf]
