# Supplementary figures and images for: Persistent immune imprinting occurs after vaccination with the COVID-19 XBB.1.5 mRNA booster in humans
Source: Immunity. Author manuscript; Available in PMC 2025 Aug 18. (PMC12360627; doi:10.1016/j.immuni.2024.02.016)

## 10 days post-vaccination

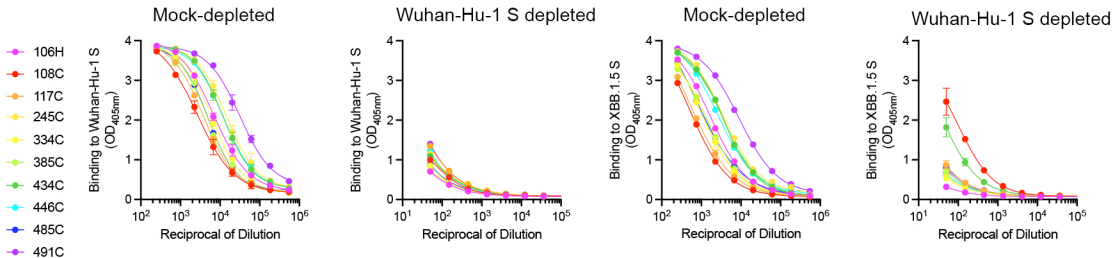

## 51 days post-vaccination

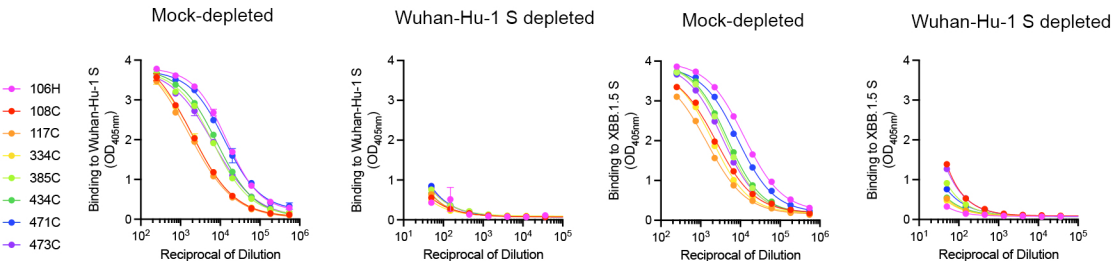

Supplement: FigS3 [file NIHMS2101333-supplement-FigS3.pdf]

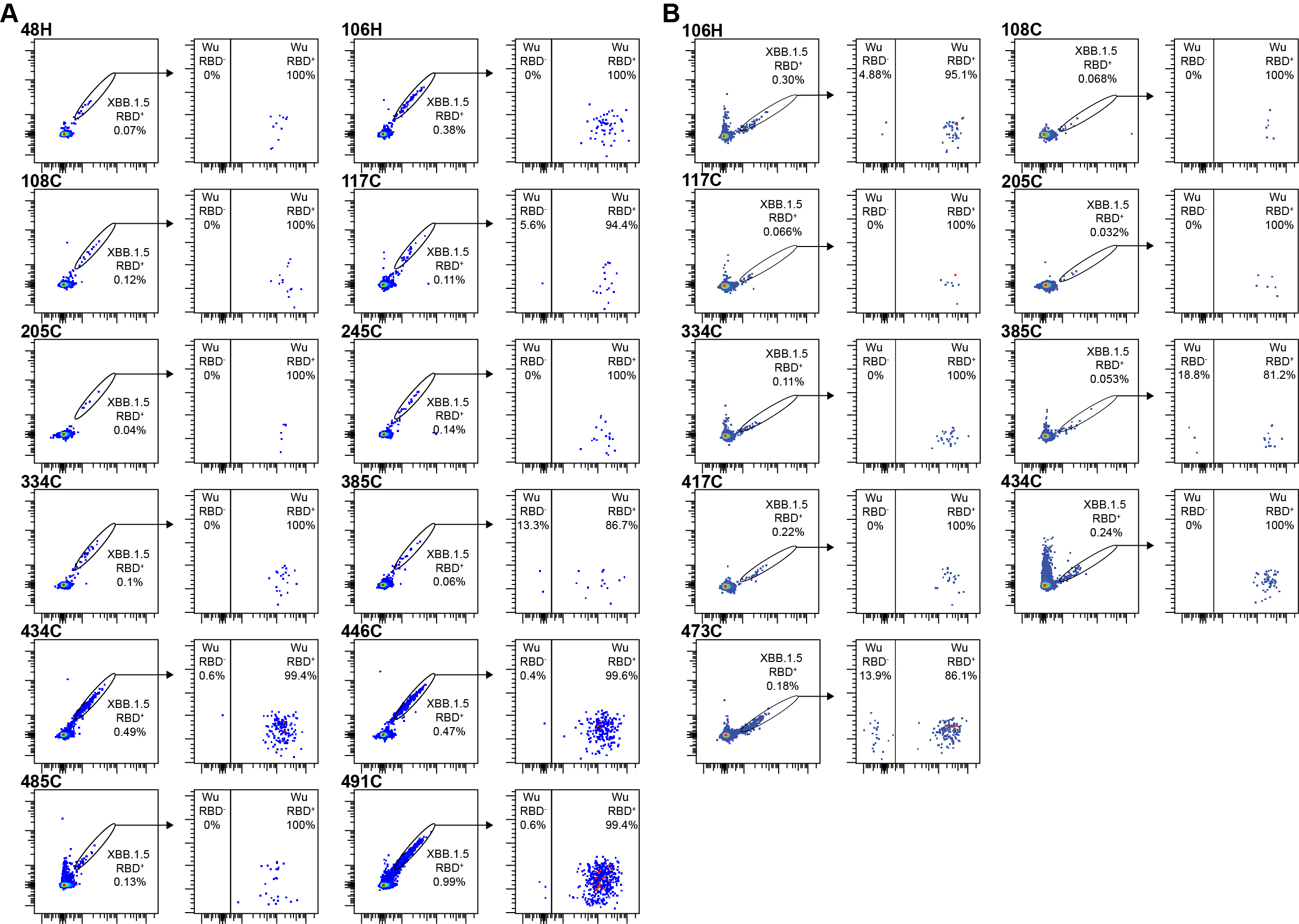

Supplement: FigS5 [file NIHMS2101333-supplement-FigS5.png]
