# Supplementary material for: Persistent immune imprinting occurs after vaccination with the COVID-19 XBB.1.5 mRNA booster in humans
Source: Immunity. Author manuscript; Available in PMC 2025 Aug 18. (PMC12360627; doi:10.1016/j.immuni.2024.02.016)
Supplement: FigS4 [file NIHMS2101333-supplement-FigS4.pdf]

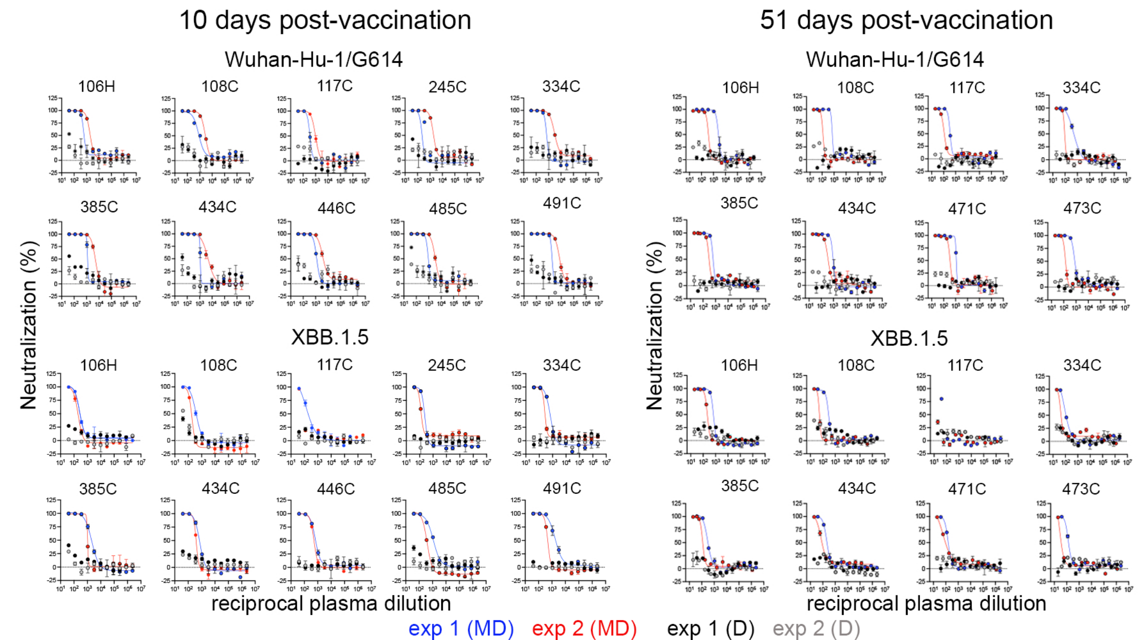
10 days post-vaccination
51 days post-vaccination
Wuhan-Hu-1/G614
106H
108C
117C
245C
334C
385C
434C
446C
485C
491C
471C
473C
XBB.1.5
Neutralization (%)
reciprocal plasma dilution
exp 1 (MD)
exp 2 (MD)
exp 1 (D)
exp 2 (D)
