# Supplementary material for: Persistent immune imprinting occurs after vaccination with the COVID-19 XBB.1.5 mRNA booster in humans
Source: Immunity. Author manuscript; Available in PMC 2025 Aug 18. (PMC12360627; doi:10.1016/j.immuni.2024.02.016)
Supplement: Supp Figure Legends [file NIHMS2101333-supplement-Supp_Figure_Legends.pdf]

Immunity, Volume *57*

Supplemental information

Persistent immune imprinting occurs after vaccination with the COVID-19 XBB.1.5 mRNA booster in humans

M. Alejandra Tortorici, Amin Addetia, Albert J. Seo, Jack Brown, Kaiti Sprouse, Jenni Logue, Erica Clark, Nicholas Franko, Helen Chu, and David Veesler

Supplemental information

Persistent immune imprinting occurs after vaccination with the COVID-19 XBB.1.5 mRNA booster in humans

M. Alejandra Tortorici, Amin Addetia, Albert J. Seo, Jack Brown, Kaiti Sprouse, Jenni Logue, Erica Clark, Nicholas Franko, Helen Chu, David Veesler

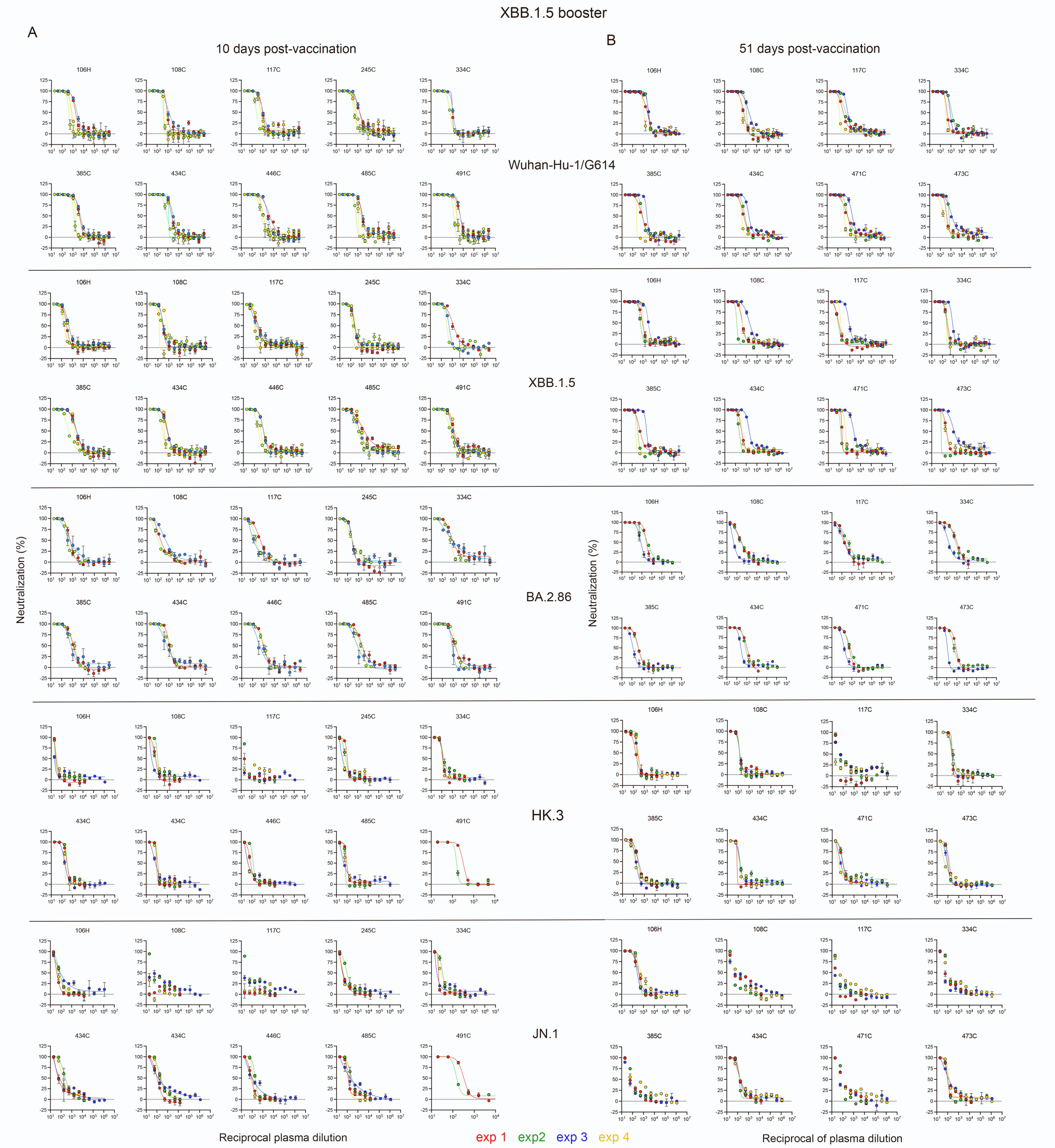

Figure S1. Plasma neutralizing antibody titers after vaccination with the XBB.1.5 S mRNA booster, related to Figure 1. (A, B). Dose-response curves for all repeats are shown with each patient ID indicated on top of each graph. Individuals 48H and 205H were not included in the analysis as their plasma did not have detectable neutralizing activity against Wuhan-Hu-1 G614 VSV. Experiments 1 and 2 (exp 1 and exp 2) were performed using a first batch of VSV S pseudotyped viruses using VeroE6-TMPRSS2 cells (puromycin resistant). Experiments 3 and 4 (exp 3 and exp 4) were performed using a second batch of VSV S pseudotyped viruses using VeroE6-TMPRSS2 cell (puromycin resistant) except for neutralizations of Wuhan-Hu-1/G614, XBB.1.5 and BA.2.86 VSV for day 10 plasma samples where experiment 3 was done using VeroE6-TMPRSS2 (geneticin-resistant) cells.

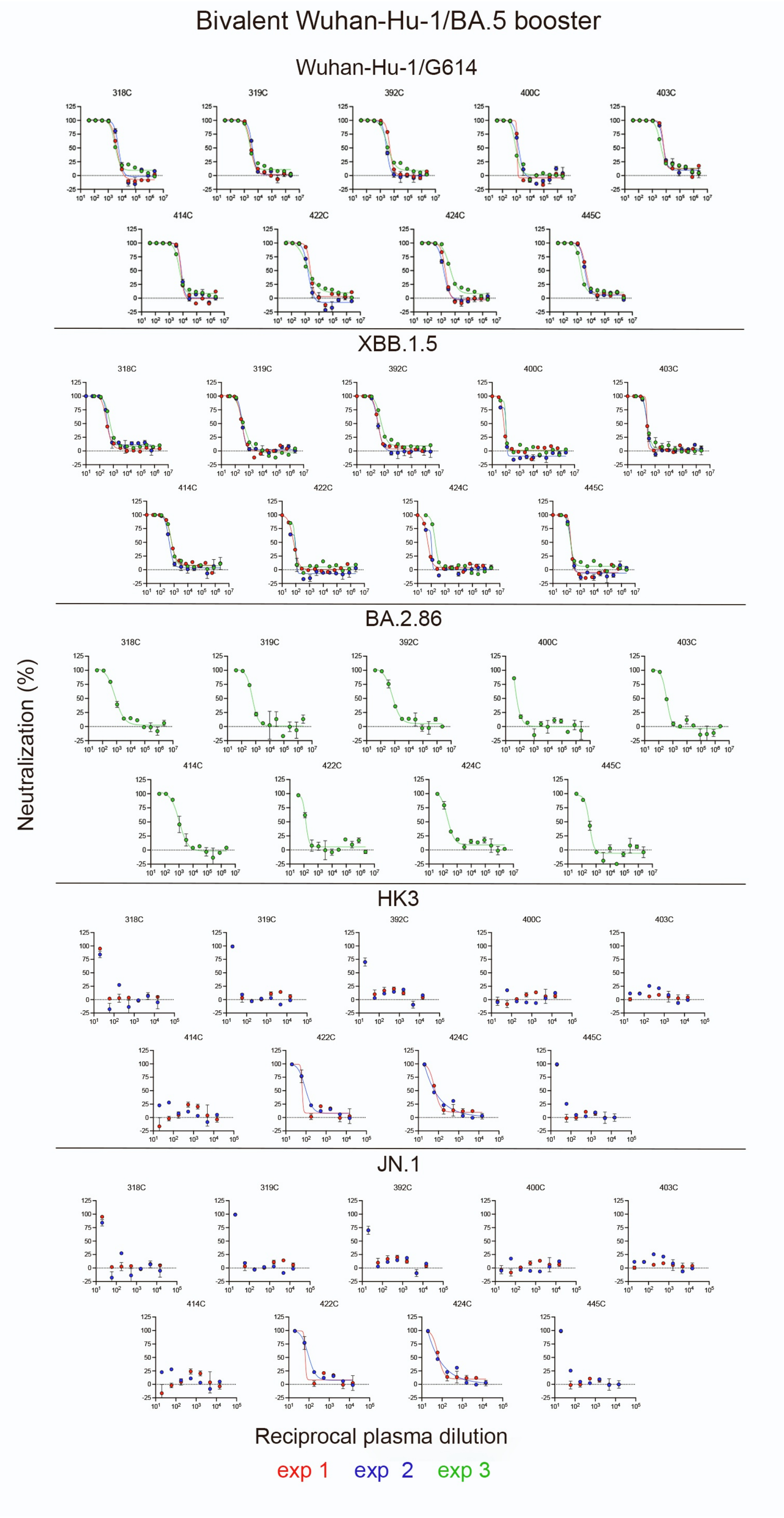

Bivalent Wuhan-Hu-1/BA.5 booster
Wuhan-Hu-1/G614
318C
319C
392C
400C
403C
414C
422C
424C
445C
XBB.1.5
318C
319C
392C
400C
403C
414C
422C
424C
445C
BA.2.86
318C
319C
392C
400C
403C
414C
422C
424C
445C
HK3
318C
319C
392C
400C
403C
414C
422C
424C
445C
JN.1
318C
319C
392C
400C
403C
414C
422C
424C
445C
Neutralization (%)
Reciprocal plasma dilution
exp 1
exp 2
exp 3

Figure S2, related to Figure 1. Plasma neutralizing antibody titers after vaccination with the bivalent Wuhan-Hu-1/BA.5 S mRNA booster. Dose-response curves for all repeats are shown with each patient ID indicated on top of each graph. Individuals 48H and 205H were not included in the analysis as their plasma did not have detectable neutralizing activity against Wuhan-Hu-1/G614 VSV. Experiments 1 and 3 (exp 1 and exp 3) were performed using a first batch of VSV S pseudotyped viruses. Experiment 2 (exp 2) was performed using a second batch of VSV S pseudotyped viruses. All the neutralizations were performed on VeroE6-TMPRSS2 cell puromycin resistant.

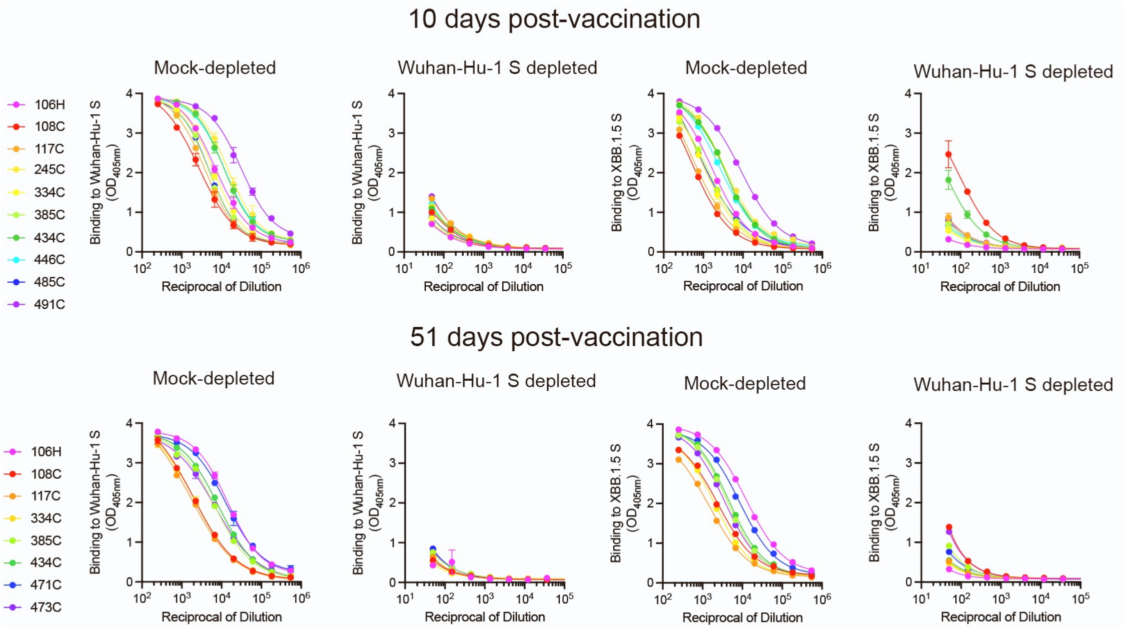

Figure S3, related to Figure 2. Antibody binding titers against Wuhan-Hu-1 S and XBB.1.5 S after vaccination with the XBB.1.5 S mRNA booster in plasma samples mock-depleted and depleted of Wuhan-Hu-1 S-reactive antibodies. Samples were collected 7-13 days (mean: 10 days, top) or 30-63 days (mean: 51 days, bottom) after receiving the XBB.1.5 S mRNA booster. Dose-response curves for one representative experiment out of 2 biological/technical replicates are shown. The color key indicates patient IDs. Individuals 48H and 205H were not included in the analysis as their plasma did not have detectable neutralizing activity against Wuhan-Hu-1 G614 VSV.

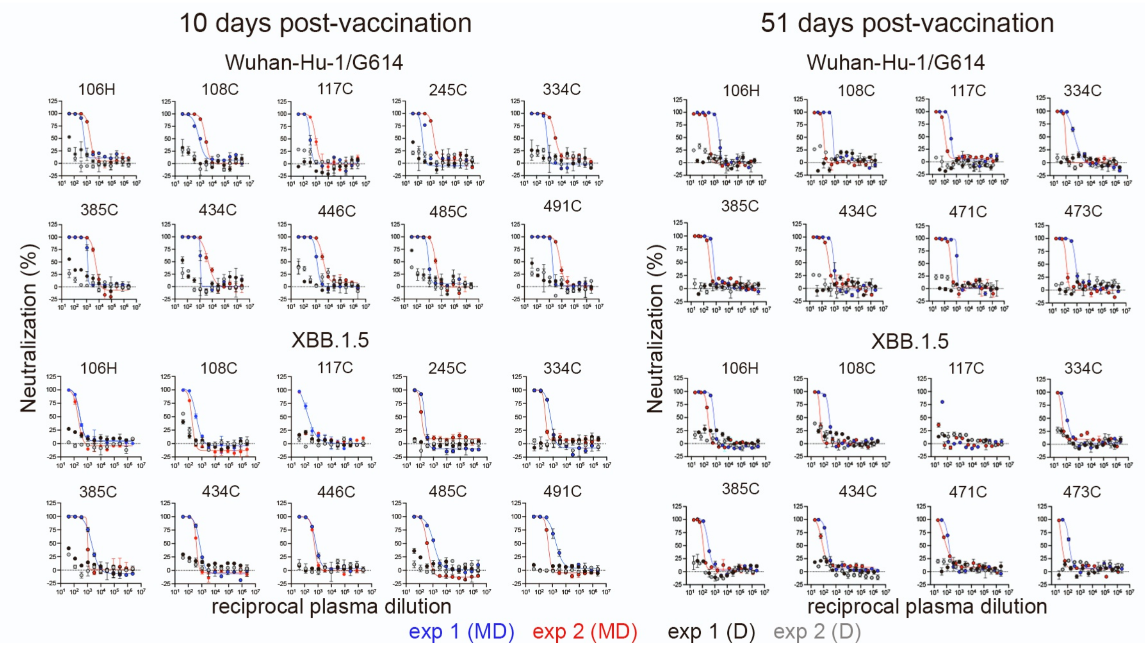

Figure S4, related to Figure 2. Neutralizing antibody titers against Wuhan-Hu-1/G614 S and XBB.1.5 S VSV after vaccination with the XBB.1.5 S mRNA booster in plasma samples mock-depleted and depleted of Wuhan-Hu-1 S-reactive antibodies. Dose-response curves of two biological experiments (exp 1 and exp 2, MD=mock-depleted and D=depleted) each performed with 2 technical duplicates. Patient IDs are indicated on top of each graph. Individuals 48H and 205H were not included in the analysis as their plasma did not have detectable neutralizing activity against Wuhan-Hu-1/G614 VSV. The plasma of individual 117C was considered to not have neutralizing activity against XBB.1.5 S VSV after mock-depletion as only one out of the two replicates had detectable (weak) neutralizing antibodies.

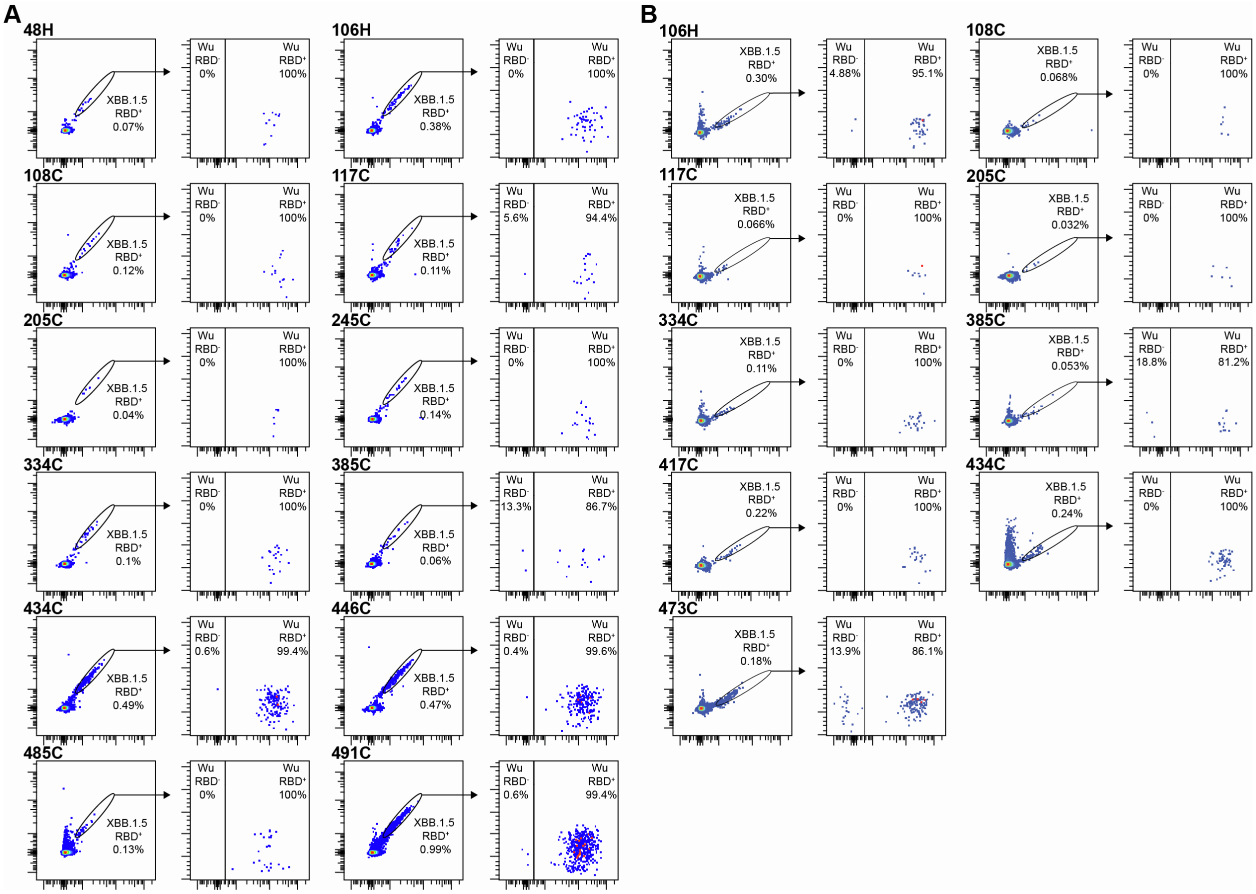

Figure S5, related to Figure 3. Flow cytometry analysis of memory B cells. Gating of XBB.1.5 RBD-reactive memory B cells and subsequent evaluation of Wuhan-Hu-1 RBD binding of these memory B from the peripheral blood of each individual collected 7-13 days (mean: 10 days, A) or 30-63 days (mean: 51 days, B) after receiving the XBB.1.5 S mRNA booster using flow cytometry. The analysis was performed once.
